# Advanced Technologies for the Extraction of Marine Brown Algal Polysaccharides

**DOI:** 10.3390/md18030168

**Published:** 2020-03-18

**Authors:** Ana Dobrinčić, Sandra Balbino, Zoran Zorić, Sandra Pedisić, Danijela Bursać Kovačević, Ivona Elez Garofulić, Verica Dragović-Uzelac

**Affiliations:** Faculty of Food Technology & Biotechnology, University of Zagreb, Pierottijeva 6, 10 000 Zagreb, Croatia; snedjer@pbf.hr (S.B.); zzoric@pbf.hr (Z.Z.); spedisic@pbf.hr (S.P.); dbursac@pbf.hr (D.B.K.); ielez@pbf.hr (I.E.G.); vdragov@pbf.hr (V.D.-U.)

**Keywords:** polysaccharides, marine algae, extraction, fucoidan, laminarin, alginate

## Abstract

Over the years, brown algae bioactive polysaccharides laminarin, alginate and fucoidan have been isolated and used in functional foods, cosmeceutical and pharmaceutical industries. The extraction process of these polysaccharides includes several complex and time-consuming steps and the correct adjustment of extraction parameters (e.g., time, temperature, power, pressure, solvent and sample to solvent ratio) greatly influences the yield, physical, chemical and biochemical properties as well as their biological activities. This review includes the most recent conventional procedures for brown algae polysaccharides extraction along with advanced extraction techniques (microwave-assisted extraction, ultrasound assisted extraction, pressurized liquid extraction and enzymes assisted extraction) which can effectively improve extraction process. The influence of these extraction techniques and their individual parameters on yield, chemical structure and biological activities from the most current literature is discussed, along with their potential for commercial applications as bioactive compounds and drug delivery systems.

## 1. Introduction

The term macroalgae refers to aquatic photosynthetic organisms which are included in the *Eukaryota* domain as well as *Plantae* and *Chromista* kingdoms [1]. They differ according to several characteristics such as cell wall composition, presence or absence of flagella and ultrastructure of mitosis [2]. Their distribution, diversity and chemical composition are mainly limited by the environmental conditions, e.g., sunlight availability (chromatic adaptation) and water temperature. Based on their pigmentation and chemical composition, macroalgae can be classified into three groups: brown (*Phaeophyceae*), red (*Rhodophyceae*) and green (*Chlorophyceae*) [3,4].

Brown algae are a rich source of bioactive molecules such as proteins, amino acids, polysaccharides, fatty acids, vitamins, minerals, dietary fibre, sterols, pigments, polyphenols etc. which possess a broad spectrum of biological activities (anticoagulant, antithrombotic, anti-viral, anti-cancer, anti-inflammatory and antibacterial) [5]. These compounds therefore provide high potential for the application of brown algae extracts in the treatment of arteriosclerosis, rheumatic processes, hypertension, goitre, asthma, ulcers, menstrual disorders, syphilis, skin diseases etc. [4,6,7]. The biological potential of brown algae is significantly contributed by polysaccharides as one of the most common and most important groups of bioactive compounds. Over the last years, considerable interest has been raised about different types of polysaccharides in brown algae cell walls, including laminarins, alginates and fucoidans which have high potential for biological applications in functional foods, cosmeceutical and pharmaceutical products [8]. The structure and composition of algal polysaccharides (APS) is determined by algae species however it is also influenced by other factors causing inter-species variation, e.g., growth location and harvesting season [8]. Vast structural variation between the APS therefore presents a challenge in terms of pre-treatments application, extraction techniques and optimization, characterization of isolated fractions and determination of their biological properties.

Chemical structure and yield of APS isolated from marine macroalgae by conventional extraction (CE) techniques can be affected by various experimental conditions (pH, time, temperature, pressure, particle size, solvent, sample to solvent ratio, agitation speed etc.). In addition, different advanced techniques such as microwave assisted extraction (MAE), ultrasound assisted extraction (UAE), pressurized liquid extraction (PLE), enzyme-assisted extractions (EAE) are assessed and applied for APS extraction [9,10,11].

In general, the chemical structure of polysaccharides determines its physical, chemical and biochemical properties as well as its biological activities [12]. Several studies have reported that their biological activity is strongly associated with their chemical structure [9]. Due to very complex mechanisms that are affected by many factors, the correlation between polysaccharide structure and biological activity is still not sufficiently clarified.

In order to improve isolation of APS, pre-treatments are usually applied to the algal biomass prior to the extraction process with the two aims: (i) to prevent co-extraction of interfering bioactive compounds with similar solubility; and (ii) to disrupt cell walls and improve mass transfer of APS into extraction solvent. The first type of pre-treatments is therefore used to remove compounds which are highly bound to the APS such as proteins, phenols and lipids, as well as mannitol and chlorophyll [13]. For that purpose, the application of various pre-treatment solvents at different temperatures has been studied. A mixture of methanol, chloroform and water at 4:2:1 (v/v/v) has been successfully used for defatting, and acetone [14] as well as mixture of acetone and ethanol [15] or methanol [16] were used to remove lipids and pigments. The second type of pre-treatments which is carried out in order to disrupt cell wall material and enhance the mass transfer of the target compounds to the extraction solvent result in an improved extraction yield [13]. In addition, several mechanical and physical methods for cell disruption which include milling, high pressure extrusion, ultrasonication and microwave pre-treatment have been described in the literature [17].

Advanced technologies may overcome some limitations inherent to CE procedures (water, acid, salt solutions) such as relatively low yields, long time, and high energy consumption and costs. The application of advanced extraction techniques such as MAE, UAE and EAE [9], as well as purification techniques (membrane separation, affinity chromatography, ion-exchange chromatography and size-exclusion chromatography) [9] has shown the potential for the recovery of APS and other marine bioactive compounds. Hence, this review presents an overview of conventional and advanced extraction techniques of marine brown APS in the latest researches done in this field. Compared to some other review articles [3,11,13] that covered similar topics, more focus is given on extraction techniques and parameters, and their influence on structural properties and biological activity of the extracted polysaccharides. Furthermore, commercial application of APS from the most current literature is also discussed.

## 2. The Chemical Structure and Bioactivity of Polysaccharides from Marine Brown Algae

Polysaccharides from marine macroalgae differ greatly from the ones present in terrestrial plants such as cellulose and starch [18]. Brown seaweed cell walls contain sulfated polysaccharides i.e., laminarin and alginate along with fucoidan, which is not present in any other type of seaweeds. These three types of APS have their own unique physical and chemical characteristics which are influenced by species, geographic location, season and population age [19].

### 2.1. Laminarin

**Figure 1:**
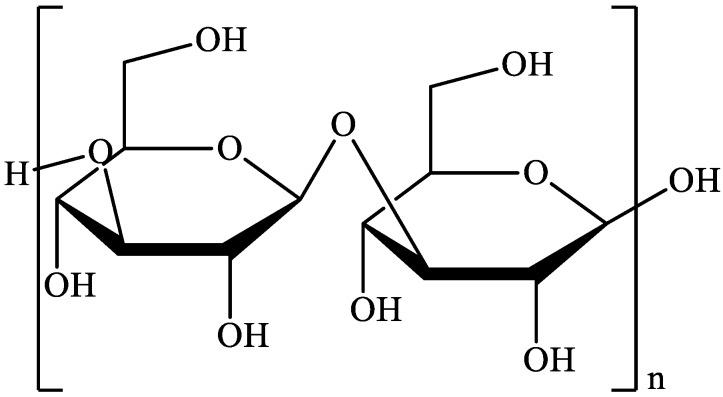
Structure of laminarin [26].

Laminarin is a water-soluble linear polysaccharide that consists of β (1→3) and β (1→6) glucan in a 3:1 ratio [20] (Figure 1). Molecular weight (MW) of laminarin is around 5 kDa depending on the degree of polymerization [21]. In addition, in dependence on the type of sugar at the reducing end, there are M chains with terminal 1-O-substituted D-mannitol, and G chains with glucose. Laminarin is mainly isolated from the brown algae species *Laminaria* and *Alaria*. Based on the type of algae and harvest season [22] as well as the environmental conditions such as sea temperature, salinity, sea currents, depth and availability of nutrients [23], laminarin represents around 22–49% of algal dry matter. Apart from contributing to dietary fibre intake, studies have shown that laminarin and products of its enzymatic hydrolysis inhibit the production of melanoma cells and colon cancer [24] and also show anti-metastatic effects which makes them potentially useful in cancer treatment [25].

### 2.2. Alginates

**Figure 2:**
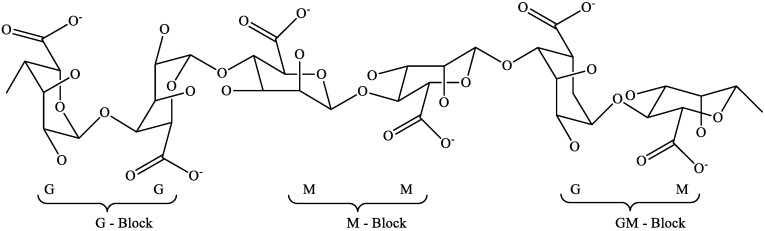
Structure of alginates [35].

Alginates are linear hetero-polysaccharides composed of β-D-manuronic acid (M) and α-L-guluronic acid (G) (Figure 2). These two monomers are linked in a 1→4 configuration and arranged as homogeneous MM, GG or alternatively MG blocks. The proportion of these three block types is responsible for the physical properties of alginates whereas alginates with high M blocks share have higher viscosity while alginates with high G blocks share have better gelling properties [27]. Alginates are obtained from cell walls of various brown algae that grow in colder seas such as *Microcystis*, *Laminaria* and *Ascophyllum* sp. [28]. In addition, alginates can be present in alginic acid form as well as in the form of its salt, which make about 40% dry matter of the algae biomass [29].

Studies have shown that alginic acid has a positive effect on preventing the absorption of heavy metals in the body, reducing blood pressure and cholesterol as well as assisting in the absorption of cholesterol [30,31]. In addition, alginates with molecular mass larger than 50 kDa showed a positive effect in prevention of diabetes and adiposity [32]. Furthermore, they have anticarcinogenic properties [31] and inhibitory effect on microorganisms such as *Staphylococcus*, *Listeria*, *Salmonella* and *Escherichia coli* [33]. Due to their properties, alginates are widely used by the food industry as stabilizers, emulsifying agent or thickeners, as well as by the cosmetic and pharmaceutical industries [34].

### 2.3. Fucoidan

**Figure 3:**
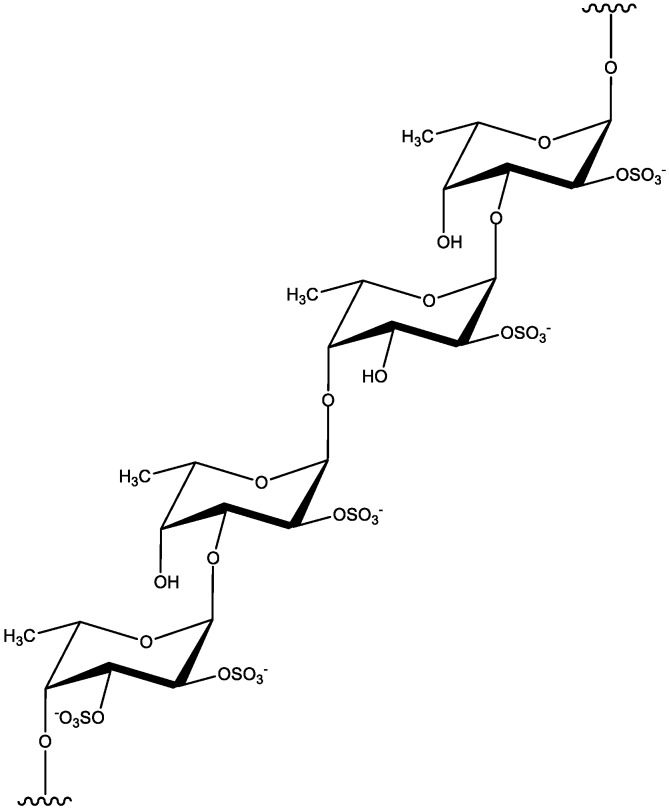
Structure of fucoidan from *Fucus vesiculosus*, with a backbone of alternating (1→3)-linked α-L-fucopyranose and (1→4)-linked α-L-fucopyranose residues and the presence of sulfate groups on both *O*-2 and *O*-3 [41].

Fucoidan is sulfated polysaccharide found in fibrillar tissue of the cell wall and intercellular space of brown algae. It consists mainly of fucose interconnected by α−(1,3) glycoside bonds, alternating α−(1,3) and α−(1,4) bonds and rarely α−(1,2) bonds (Figure 3). Apart from fucose, it also contains other monosaccharides, including galactose, glucose, mannose, xylose, rhamnose, and uronic acids which contents vary depending on algal species and season [19,36]. The average relative MW of fucoidan varies from 7 kDa [37] to 2300 kDa [38]. Fucoidan is the most abundant in orders *Laminariales* and *Fucales* and, depending on the algae type, it represents approximately 5% to 10% of algae dry matter while its sulfate content varies between 5% [19] and 38% [39].

Fucoidan is one of the most researched algae molecules and studies have found that it shows a wide range of positive effects such as antioxidant, anti-inflammatory and antitumor [10,19,40]. It has been increasingly studied not only for its potential applications as the heparin-like anticoagulant and antithrombotic agent but also, due to its non-toxicity and biodegradability, as a functional additive for developing novel drug delivery systems and functional foods. Biological activities of fucoidan are closely correlated to its physicochemical properties such as MW, types and ratios of constituent monosaccharides, features of glycosidic linkages, sulfation degree and the position of sulfate groups.

## 3. The Perspective of Advanced Technologies for Polysaccharide Extraction from Marine Brown Algae

**Figure 4:**
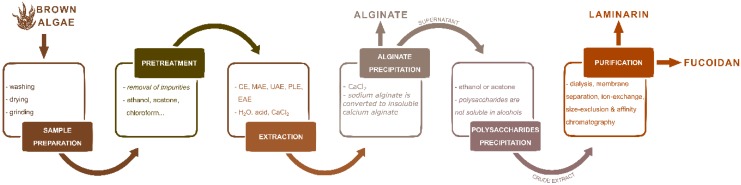
Schematic overview of essential steps for extraction of brown algae polysaccharides.

In general, APS isolation process (Figure 4) includes several complex and time consuming steps such as seaweed preparation, pre-treatment, extraction (conventional or advanced) and purification. These procedures are usually followed by biological activity assays which enable the determination of APS potential industrial use.

**Figure 5:**
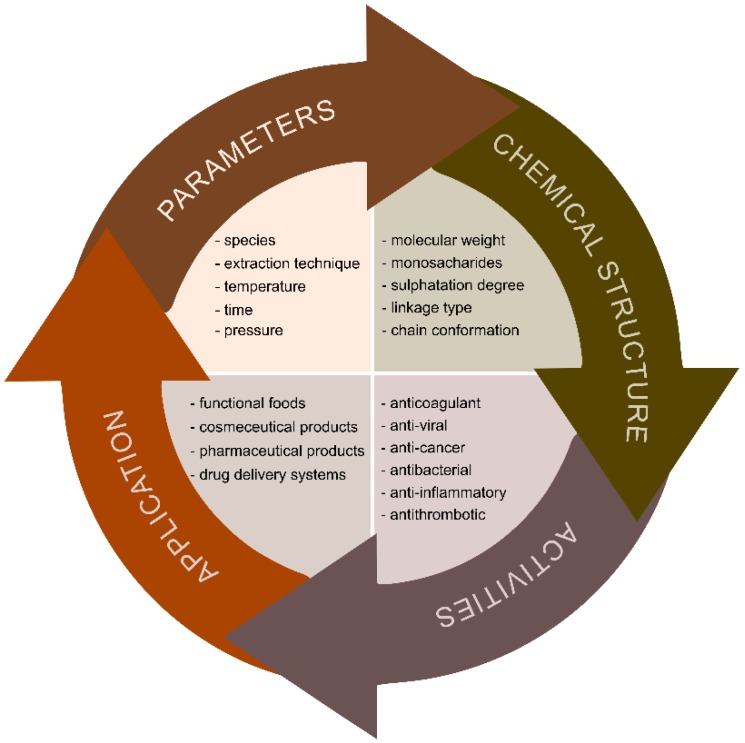
Schematic diagram of process parameters, chemical structure properties, biological activity and potential industrial uses of brown algae polysaccharides.

Preparation of the seaweed includes washing the seaweed, preferably with distilled water to remove salt and impurities, as well as drying or freeze drying and milling in order to achieve the powder with higher surface-to-volume ratio. Removal of interfering algal compounds prior to polysaccharide extraction is useful for prevention of contamination of the target polysaccharide. Conventional polysaccharide extraction is usually performed by hot aqueous or acidic solutions at high temperatures for several hours. On the other hand, advanced technologies (e.g., MAE, UAE, PLE and EAE) have already demonstrated numerous advantages over CE especially in the terms of increased extraction efficiency, reduction of extraction time, energy and solvent usage as well as preservation of sensitive and unstable bioactive molecules (e.g., polyphenols). Regardless of which extraction technique is performed, the extraction parameters should be optimized as they may influence APS chemical structure, bioactivity and potential industrial use (Figure 5). Moreover, preservation of the APS structural integrity is crucial for obtaining the relevant structural characteristics required for their specific biological activities [10].

### 3.1. Pre-treatment of Marine Brown Algae

Prior to the APS extraction, it is beneficial to apply pre-treatment in order to remove lipids, pigments and low molecular weight compounds from the seaweed material. For that purpose, various solvents and solvent mixtures with different polarity, that do not cause any APS structural changes, have been used [13]. Lipids are traditionally removed by lower polarity solvents, such as chloroform, petroleum ether or dichloromethane; pigments with semipolar solvents, such as acetone, methanol or ethanol; while other high polarity molecules, such as monosaccharides, proteins and minerals are extracted in water [20]. Prior to laminarin extraction from *Cystoseira barbata*, Sellimi et al. [42] seaweed powder was sequentially treated twice with acetone-methanol (7:3) and twice with chloroform for 24 h at 30 °C with constant stirring (250 rpm). Similarly, January et al. [43] used the mixture of methanol-chloroform-water (4:2:1) as a pre-treatment prior to fucoidan extraction. However, as chloroform is toxic and classified as an extremely hazardous substance in the United States as it is defined in Section 302 of the U.S. Emergency Planning and Community Right-to-Know Act (42 U.S.C. 11002), new chloroform-free pre-treatment alternatives are being proposed. As an alternative solvent, relatively low cost and non-toxic petroleum ether has been used by Sahera et al. [44]. Furthermore, *Cystoseira myrica* powder was treated with petroleum ether and acetone in Soxhlet apparatus prior to polysaccharides extraction. The same procedure was applied by Abid et al. [45] for sodium alginate extraction from *Dictyopteris membranaceae* and *Padina pavonica*. However, in their work, algae powder was previously macerated three times by methanol and dichloromethane (1:1, v/v) for 48 h. In addition to acetone, 95% ethanol (v/v), 80% ethanol (v/v) and methanol were also used [15,16,46]. Compared to previously described procedures, where higher temperatures were not used because they might promote the extraction of fucoidan, with 80% ethanol (v/v) as a solvent, higher temperature is acceptable. Since fucoidan is not soluble in ethanol, even if it was unintentionally extracted, it would be precipitated and thus only impurities like pigments and lipids would be removed by filtration [19].

Prior to APS extraction, pre-treated seaweed is further exposed to the conventional, vacuum or freeze drying methods. In addition, as seaweed rigid cell walls are hard to break [47], a cell disruption pre-treatment is generally required to remove or weaken them, making the intracellular molecules more accessible to solvent in further extraction steps. For that purpose, various cell disruption pre-treatment methods such as mechanical, chemical, thermal, enzymatic or advanced techniques, like ultrasound and microwave, could be applied. Ultrasound and microwave pre-treatments are based on the energy waves that have an effect on the cell wall material causing its lysis and release of intracellular molecules. However, this approach is more often used in bioethanol production from algae where pre-treatment is intended for cell disruption and complex carbohydrates release. Complex carbohydrates are then broken down into their monosaccharide components (simple sugars) which are fermented into bioethanol and carbon dioxide [48]. To the best of our knowledge, the only research where cell disruption pre-treatment was applied prior to polysaccharide extraction from brown seaweed was made by Kadam et al. [49]. In their work, ultrasound pre-treatment for 10 min (20 Hz, amplitude 20–100%, 13 mm diameter probe) was applied followed by simple extraction in orbital shaker for 1 to 22 hours to extract fucose and uronic acid from *Ascophyllum nodosum*. Compared to control extraction, ultrasound pre-treatment increased fucose and uronic acid extraction yield, whereas ultrasound amplitude was the most significant factor contributing to the pre-treatment efficiency [49].

### 3.2. Extraction Techniques

Following pre-treatment, seaweed samples are subjected to various extraction techniques. General principal of these procedures is to extract target compounds with minimum co-extraction of other polysaccharide constituents, e.g., isolate fucoidan from alginate. If alginate is co-extracted, further steps are needed to remove alginate from fucoidan, thus increasing the purity of the extracted fucoidan [20].

#### 3.2.1. Conventional Extraction Technique (CE)

Conventional extraction of APS is typically performed by treating the algal material with various solvents such as hot water, acidic or salt solutions at high temperatures for several hours. Table 1 summarizes the most frequently used parameters of CE for APS recovery from brown algae.

CE with hot water (80–100 °C) is often used to extract water-soluble sulfated APS from *Sargassum henslowianum* and *Dictyopteris divaricate* [46,50]. However, this method is not sufficiently selective as all types of APS (fucoidan, alginate and laminarin) and other water-soluble compounds from the seaweed samples could be also extracted. Consequently, more isolation steps are needed to increase the purity of fraction with target polysaccharide.

Furthermore, for the improvement of the extraction yield, the use of 0.1 M HCl solution has been shown to be effective since it enables cell walls hydrolysis and facilitates fucoidan and laminarin extraction from the matrix [42,51]. In addition, the acid converts alginate into water-insoluble alginic acid, which is removed, together with solid seaweeds residues, resulting in relatively pure fucoidan fraction [20]. For alginate extraction, solid residue remaining after water and acid extraction could be treated with sodium carbonate (Na_2_CO_3_) to convert alginic acid into sodium alginate, which is water-soluble but not alcohol-soluble. Therefore, by the addition of ethanol, formed sodium alginate can be precipitated, separated from the rest of the mixture and dried [16,52].

To effectively remove alginate which is present in brown algae cell walls in the form of calcium, magnesium and sodium salts of alginic acid, 2% calcium chloride (CaCl_2_) solution is often used [15,45,53]. Since only sodium salt is water soluble, aqueous solution of CaCl_2_ enables fucoidan and sodium alginate extraction and dissolution, while high temperature and mechanical agitation additionally enhance the extraction process. However, when sodium alginate gets in contact with calcium ions, they replace the sodium ions in the polymer and solid calcium alginate is formed. It is not soluble in water and can be easily separated, leaving relatively pure fucoidan in the extract.

January et al. [43] used all three previously described solvents (salt - CaCl_2_, acid and water) to extract fucoidan from *Ecklonia maxima*, *Laminaria pallida* and *Splachnidium rugosum*. Their results showed that conventional hot water extraction (HWE) resulted in the highest concentration of L-fucose while acid extraction resulted in the highest sulfate and uronic acid content. On the contrary, while investigating fucoidan extraction from *Sargassum fusiforme*, Liu et al. [54] achieved the lowest sulfate and uronic acid content by applying acid as a solvent. Furthermore, they achieved the highest fucoidan yield with acid extraction (11.24%) and the lowest with CaCl_2_ method (3.94%). MW of fucoidan extracted with acid was significantly lower while acid and salt extraction removed almost all protein indicating higher purity of the extract. DPPH and hydroxyl radical scavenging activities were much higher for fucoidan extracted with water and salt compared to acid extracted fucoidan which was positively correlated with the uronic acid content, MW and monosaccharide composition (glucose + galactose).

#### 3.2.2. Advanced Extraction Techniques

Recently, advanced extraction techniques used for polysaccharide extraction from algae are microwave-assisted extraction (MAE), ultrasound assisted extraction (UAE), pressurized liquid extraction (PLE) and enzymatic assisted extraction (EAE). However, due to various extraction conditions, APS degradation could occur which may affect the extract viscosity, sulfate content, monosaccharide composition, MW and bioactivity. Therefore, extraction parameters such as temperature, time, power and sample to solvent ratio should be optimized.

##### Microwave Assisted Extraction (MAE)

MAE is considered one of the most efficient extraction techniques that can overcome drawbacks of conventional procedures. During microwave treatment, heat is generated directly within the material (volumetrically distributed heating) by ionic conduction of dissolved ions and/or dipole rotation of polar solvent. Non-polar compounds are hence not heated when exposed to microwaves. Rapid internal heating during MAE causes an effective cell wall rupture and release of the intracellular compounds into the extraction solvent [55]. Microwave radiation can also stimulate cuticular layer destruction which was observed as very rough algae surface with many cavities after high pressure (120 psi) MAE application [56]. MAE has been successfully used for isolation of various bioactive compound from seaweeds [57,58,59,60] as well as polysaccharides from other plants [61,62,63]. Application of MAE for brown APS extraction is summarized in Table 2 along with its effect on chemical structure and bioactivities of target polysaccharides.

Alboofetileh et al. [64] confirmed that MAE is more efficient than conventional HWE of polysaccharides from *Nizamuddinia zanardinii* with extraction yields being 6,17% vs. 5,2%, respectively. Higher crude extract yield can be correlated to higher amount of bioactive compounds, but there is a possibility that it is a result of higher amount of impurities [65] which can explain higher yields obtained by CE compared to MAE in other research [55,65]. These contradictory results may be attributed to differences in extraction protocols, algae species, origin, harvest time and extraction conditions [65]. Another point that should be taken into consideration when considering method efficiency is much shorter extraction time of MAE as well as 3 times lower solvent volume [55].

Polysaccharides obtained by MAE have higher concentration of sulfate groups and lower MW [65,66]. Alboofetileh et al. [64] reported higher sulfate content and higher MW while Yuan and Macquarrie [55] reported lower sulfate content and lower MW of the polysaccharides extracted by MAE compared to HWE. MAE had no significant effect on type of glycosidic bond and monosaccharide compositions in fucoidan from *Sargassum thunbergii* [66] while higher fucose content was reported for *A. nodosum* and *N. zanardinii* fucoidan [55,64,65] as well as lower uronic acid content [55,64,65]. On the contrary, higher uronic acid, galactose and neutral sugars contents was reported by Okolie et al. [65]. Moreover, polysaccharide extracts obtained by MAE showed higher antioxidant and hydroxyl radical scavenging activity as well as potential hypoglycemic activity [66] due to the lower MW and higher sulfate groups content in comparison to CE. They also improved the growth rate of *Lactobacillus delbruecki* ssp *bulgaricus*, while no apparent effect was found on the growth rates of *Lactobacillus casei* [65]. Fucoidan extracted by MAE at 90 °C had similar DPPH scavenging effect and even higher reducing power than fucoidan extracted by CE [55]. At 2 mg/mL MAE polysaccharides inhibited the growth of *Escherichia coli* although showed lower inhibitory activity against HSV-2 strain [64].

Besides extraction technique, extraction parameters such as microwave power, irradiation time, temperature and pressure also have an impact on polysaccharide yield and thus should be optimized. Polysaccharides extraction yield from *Sargassum pallidum* and *S. thunbergii* increased significantly with increased time, temperature and power, while optimal MAE conditions were set at 10 min, 90 °C, 800 W [67] and 20 min, 70 °C, 600 W [66]. Similar trend for time and temperature influence was observed by Yuan & Macquarrie [55] on *A. nodosum* polysaccharides, where the highest yield was achieved at 120 °C for 15 min. It is expected that an increase in temperature should decrease the viscosity and surface tension therefore improving compound solubility, diffusion rate and mass transfer in the solvent [68]. In the initial extraction stage higher temperature, longer time and higher power accelerated the mass transfer of intracellular substances. However, excessive extraction time, temperature and microwave power can lead to the degradation of some APS and reduced yield. As shown by Rodriguez-Jasso et al. [56] interaction between pressure and extraction time in MAE was highly significant (*p* < 0.01) for fucoidan yield from *Fucus vesiculosus* and maximum yield (18.22%) was achieved when the highest pressure (120 psi) and the lowest extraction time (1 min) were applied.

MAE parameters also had a strong influence on fucoidan monosaccharide composition, sulfatation degree, MW and biological activities. The monosaccharide composition of fucoidan from *A. nodosum* indicated that fucose is the major constituent of fucoidan extracted at 90 °C while glucuronic acid is the main component of fucoidan isolated at 150 °C [55]. Similarly, by increasing the extraction pressure from 30 psi to 120 psi fucose content, in fucoidan from *F. vesiculosus*, decreased from 100% to 27% and galactose content increased from 0% to 57% [56]. In addition to sulfatation degree of fucoidan that decreased with the increase of extraction temperature during MAE [55,56], scavenging effect on DPPH free radicals and reducing power also decreased with the increase in temperature and time [55]. Sulfate groups could contribute to the hydrogen-donating ability of the polysaccharides by activating the hydrogen atom of the anomeric carbon [69]. Therefore, increased sulfatation degree observed during these MAE experiments could potentially increase antioxidant, anticoagulant and anti-HIV activity of extracted APS [55].

##### Ultrasound Assisted Extraction (UAE)

Among the novel techniques, UAE is the most practical for the industrial level because of its simplicity, faster extraction rate, increased yield as well as reduced cost and processing time [70]. UAE can also be combined with other non-conventional technologies, such as enzymatic processing [71] or MAE [64]. The acoustic cavitation in UAE generates physical forces such as shear, shockwaves, micro jets and acoustic streaming [72], causing cell walls disruption, particle size reduction and better contact between solvent and target compounds. Furthermore, ultrasound causes a rapid formation and collapse of cavitation bubbles in treated liquid medium, leading to intense stress and irreversible chain splitting [73]. Ultrasound treatment could cause structural (MW, monosaccharide compositions, sulfate content) and microstructural modifications of the sulfated APS. Alboofetileh et al. [71,74] examined surface microstructure of fucoidan with scanning electron microscope (SEM) and micrographs showed fucoidan as distributed fluffy powder under 200 fold magnification and an irregular semi-spherical shape with no uniform size and plenty of pores at 500 and 1000 fold magnification. UAE efficiency is dependent on various factors, such as ultrasound power, temperature, time, solvents to solids ratio and characteristics of the compounds to be extracted, hence optimization of the extraction conditions is important.

Improved yield achieved by UAE in comparison to CE is attributed to the bubble cavitation phenomenon generated by ultrasonic waves [75], that was previously observed in fucoidan extraction from *Sargassum witghtii* [70], *Undaria pinnatifida* [76] and laminarin extraction from *A. nodosum* and *Laminaria hyperborean* [75]. No statistical difference between UAE and CE was reported in fucoidan extraction from *N. zanardinii* [64] and *Fucus evanescens* [77], while Okolie et al. [65] reported significantly higher yield by CE (11.9%) than by UAE (4.56%) and also no statistical differences between UAE, MAE and EAE. Alboofetileh et al. [74] compared UAE, EAE and combined UAE-EAE for isolation of polysaccharides from *N. zanardinii*. Extraction technique had a strong impact on fucoidan yield - the lowest yield was obtained by UAE (3.6%) while alcalase enzyme improved disintegration of cell wall, hence UAE-EAE exhibited the highest yield (7.87%).

Temperature controlled UAE equipment enables the study of temperature impact on polysaccharide yield. The effect of temperature in range of 30–90 °C and 70–90 °C was studied by Youssouf et al. [78] and Alboofetileh et al. [71], respectively. They noted that extraction yield increased linearly with temperature increase and reached maximum at 90 °C. By increasing the temperature, surface tension and solvent viscosity are reduced and vapour pressure is increased. Therefore, it is easy to form cavitation bubbles which intensify cellular damage, promote intracellular polysaccharides extraction and improve extraction yield [79]. By increasing the ultrasound power from 75 W to 150 W, extraction yield of alginates from *Sargassum binderi* and *Turbinaria ornata* also improves [78]. Similarly, fucoidan yield from *N. zanardinii* increased by increasing the power from 50 W to 200 W but slightly decreased above 200 W [71]. It was previously established that ultrasonic power facilitates cell walls breakdown and APS diffusion into the solution. However, greater ultrasound power could lead to chemical decomposition caused by hydroxyl radicals generated by acoustic cavitation [79]. Fucoidan and alginate yield increased linearly with increased extraction time until they reached plateau at 40 and 30 min respectively [71,78]. Youssouf et al. [78] noted positive correlation between pH and alginate yield since high pH leads to the formation of water soluble sodium alginate.

Regarding sulfate content there was no unique trend among different authors, obtained by UAE in comparison with CE, since some reported higher sulfate concentration obtained by UAE [71], some similar [70] and some even lower [65]. Fucoidan extracted by UAE (22.97%) had lower sulfate content than the one extracted by EAE (29.6%) but higher than UAE-EAE (21.78%) [74]. In majority of extracted fucoidans main monosaccharides were fucose, mannose, galactose, xylose and glucose, with no noted impact of the extraction technique used. By comparing UAE and CE, Okolie et al. [65] reported that there was no significant difference for fucose and galactose concentration. Alboofetileh et al. [64] found no significant difference in fucose and uronic acid concentration but galactose content was slightly increased by UAE. However, destructive effect of ultrasound on fucose structure caused slight reduction of fucose concentration in UAE (23.72%) compared to HWE (26.21%) [70]. UAE demonstrated to be more efficient in extraction of higher MW fucoidan [64,65,71] and laminarin [75] then CE, MAE, PLE, EAE and UAE-EAE. Nevertheless, UAE reduced average MW of the *U. pinnatifida* polysaccharides [77].

Considering antioxidant activity of APS extracts, Hanjabam et al. [70] reported lower DPPH radical scavenging activity and reducing power along with higher metal chelating activity of fucoidan extracted by UAE in comparison to CE. Laminarin extracted from *L. hyperborea* and *A. nodosum* by UAE using acid as a solvent had the highest DPPH activity, 87.58% and 93.24% respectively [75]. Furthermore, anticancer activity of fucoidan extracted by UAE was lower than the one extracted by EAE and UAE-EAE [74]. Even though sulfated polysaccharides extracted from *N. zanardinii* by UAE didn’t show antibacterial activity they exhibited potential anti-HSV-2 activity, with EC50 value of 0.082 µg/mL compared to 0.031 µg/mL in HWE [64]. Table 3 summarizes the most frequent used parameters of UAE for APS recovery from brown algae.

##### Pressurized Liquid Extraction (PLE)

PLE is novel extraction technique based on using elevated temperatures and pressures to extract compounds from samples in oxygen and light-free environment, in a short period of time and using less solvent. Elevated temperature allows the sample to become more soluble and achieves a higher diffusion rate, while elevated pressure keeps the solvent below its boiling point [80]. Depending on the solvent used for the extraction and its diverse working conditions, PLE is often called pressurized fluid extraction (PFE), pressurized solvent extraction (PSE), accelerated solvent extraction (ASE), subcritical water extraction (SWE) or hot water extraction (HWE) [80]. For polysaccharide extraction from brown algae different type of static [81] or dynamic [82,83,84,85] PLE equipment has been used. Commercial laboratory-scale equipment commonly used for PLE was developed by the Dionex Corporation in 1995 and it can only be operated in static (batch) mode while some in-house equipment can be used in dynamic mode (continuous flow) [86].

High temperature (>100 °C) and pressure (> 10 MPa) modify physical properties of solvent that improves its penetration, capillary effects and cell destruction, resulting in increased fucoidan yield of water PLE (13.15%) compared to CE (5.2%) from *N. zanardinii* [64]. Along with improved yield, extraction time was reduced from 6 h (two cycles of 3 h) to 20 min (two cycles of 10 min). Likewise, fucoidan yield from *N. zanardinii* obtained by PLE under optimized conditions (29 min, 150 °C, water to sample ratio of 21 mL/g and pressure of 7.5 bar) was 25.98% which was considerably higher than 5.2% obtained by CE [87]. Saravana et al. [85] achieved almost 2 times higher fucoidan yield from *Saccharina japonica* by PLE under optimized conditions (11.98 min, 127.01 °C, 80 bar and 0.04 g/mL) than by CE, while *S. japonica* extraction yield was more than 4-times higher by PLE (140 °C, 50 bar) with 0.1% NaOH as a solvent compared to 0.05M HCl CE [82].

Even though PLE caused lower APS sulfate content in comparison to HWE, MAE and EAE [64], sulfate content achieved by using PLE with 0.1M NaOH as solvent was almost 2-folds higher than in CE [85]. In both of these studies uronic acid and fucose content were higher, while galactose and glucose content obtained by PLE were lower. Additionally, Saravana et al. [85] obtained higher total sugar, protein and phenolic content by PLE, meaning that besides improved yield impurities were also increased. Polysaccharides obtained by PLE had lower MW and higher polydispersity [64,82,85]. In contrast to polysaccharides obtained by CE, PLE extracted polysaccharides showed antibacterial activity and antiviral activity against HSV-2 infection [64]. Moreover, fucoidan obtained by PLE also showed good antioxidant, modest anti-mitotic and moderate anti-proliferative activities in cell lines [85]. There was no significant difference in DPPH radical scavenging activity between CE and PLE with water, 0.1% NaOH and 0.1% formic acid as solvents while ABTS^+^ radical scavenging activity was significantly higher for PLE extract obtained with 0.1% formic acid and water then for CE extract [82]. Hydrogen atoms from different monosaccharide components and their side-chain linkages may be the reason for scavenging capacity of polysaccharides extracted with water and NaOH [82].

Temperature plays a key role with respect to fucoidan yield. In general, elevated temperature results in improved extraction yield [83,87] up to a certain point after which yield stagnates or decreases [82,85]. Similar effect occurs with UAE and has been previously explained. A group of authors [83,85] determined polysaccharides yields from *Saccharina japonica* hydrolysates and found them to be enhanced by increasing the pressure from 20 to 80 bar [85] and from 13 to 520 bar [83]. Similarly, polysaccharide yield increased when extraction time was increased from 10 to 30 min in the research of Alboofetileh et al. [87]. Sample to solvent ratio was the main factor affecting the fucoidan yield in the research by Saravana et al. [85]. In a tested range from 0.04 to 0.09 g/mL yield was enhanced up to 0.05 g/mL and decreased with further sample to solvent ratio increase. RSM and Box–Behnken design (BBD) were used to determine optimal conditions for fucoidan extraction from *N. zanardinii* [87] which were determined at the temperature of 150 °C, time of 29 min and sample to solvent ratio of 21 g/mL. Temperature of 127.01 °C, pressure of 80 bar and sample to solvent ratio of 0.04 g/mL were optimal conditions for fucoidan extraction from *S. japonica* [85].

By using 0.1% NaOH, 0.1% formic acid and water as extraction solvent, higher temperature positively influenced the sulfate content, although temperatures higher than 170 °C under the pressure of 75 bar did not contribute to further yield increase [82]. Fucoidan extracted with 0.1% formic acid had the lowest sulfate content followed by water extract and 0.1% NaOH extract. Ester bonds between polysaccharide chain and sulfate groups are not easily broken by NaOH while water at high temperature breaks down ester bonds more effectively [82]. The highest uronic acid content in extracted fucoidan was obtained by using formic acid at 110 °C and 25 bar, while the highest total sugar concentration was obtained at 140 °C and 50 bar [82] and at 180 °C and 13 bar [83]. As temperature and pressure further increased, concentration of uronic acid and sugars gradually decreased indicating that monosaccharides are not stable at higher temperature and pressure [83]. Fucose was the main monosaccharide of the fucoidan extracted by PLE, while mannose, galactose, xylose and glucose were also present in the majority of samples [81,82,87]. MW of fucoidan extracted with formic acid was significantly lower than that of fucoidan extracted with 0.1% NaOH, water or ethanol, indicating that acid extraction may have caused APS chains decomposition [82]. Saravana et al. [84] used subcritical water treatment to depolymerize fucoidan powder extracted from *U. pinnatifida* and to study the influence of different parameters on the antioxidant activity (DPPH and ABTS^+^). Results showed that antioxidant activity is increased as temperature and pressure are increased for both DPPH and ABTS^+^, however after 250 °C the activity was reduced. Table 4 summarizes the most frequently used parameters of PLE for APS recovery from brown algae.

##### Enzymes Assisted Extraction (EAE)

Enzymes assisted degradation of cell wall polysaccharides is a useful technique widely used to improve extraction efficiency of bioactive compounds from terrestrial plants but not that often from seaweed. EAE showed higher extraction yield, faster extraction rate, lower energy consumption and simpler recovery with reduced solvent usage in comparison to CE [88]. EAE of polysaccharides could be performed with enzymes capable of cell wall degradation or enzymes that cause partial degradation of desirable polysaccharides into smaller fragments with the aim of facilitating the extraction. Various types of commercially available carbohydrate hydrolytic enzymes and proteases are used for APS extraction. Their use is practical in commercial applications and cost effective for industry, whereas seaweed polysaccharide-specific hydrolytic enzymes such as fucoidanases and alginases are still difficult to access [89]. Since fucoidans are closely associated with cellulose and proteins, which limit their extractability by chemicals, their hydrolysis by commercially available carbohydrate hydrolytic enzymes and proteases can facilitate weakening of the cell wall complex and release of the target APS (fucoidan and alginate) without significant degradation [89]. Some of the commercially available enzymes are: (i) Alcalase—protease which hydrolyse peptide bonds; (ii) Viscozyme—a mixture of carbohydrases (cellulase, β-glucanase, hemicellulase and xylanase) that catalyzes the breakdown of pectin-like substances in the algal cells; (iii) Celluclast—able to break down cellulose in algal cells into glucose, cellobiose and longer glucose polymers; (iv) AMG—a type of amyloglucosidase which breaks down starches consisting of 1,4 and 1,6 linkages; (v) Termamyl—a type of heat-stable α-amylase; and (vi) Ultraflo—a type of multiactive β-glucanase [18]. Apart from the enzyme type other process parameters such as temperature, time, pH and enzyme concentration or enzyme to sample ratio, are crucial for the extraction process and should be optimized.

Results demonstrated by Alboofetileh et al. [90] showed better cell wall disruption by Alcalase. Higher extraction efficiency of fucodian (5.58%) was therefore obtained with enzymatic assistance compared to conventional HWE (5.2%). Furthermore, fucoidan yield obtained by the use of Celluclast (4.8%) and Viscozyme (4.28%) was lower than the one obtained by CE. The reason for that could be that polysaccharides were probably partially hydrolysed after prolonged extraction time (24 h) in the presence of the enzymes. The lowest extraction yield of alginate (3.30%) was achieved with water extraction while slight increase was observed after alcalase (3.5%) and cellulase (3.47%) treatments [91]. Similarly, the yield of sulfated polysaccharides from *Turbinaria turbinate* obtained from cellulose, amyloglucosidase and vicozyme assisted extraction were higher than those obtained without EAE processes [92]. Alginate yield increased up to 6.60% after Cellulase treatment while Alcalase did not improve alginate yield in comparison with conventional water extraction without enzyme assistance (3.8%) [93]. Total sugar yield and composition was differently affected by carbohydrate hydrolases (Viscozyme, Celluclast and Ultraflo) and proteases (Alacalase, Neutrase and Flavourzyme) [89]. Viscozyme and Celluclast produced around twice as much total sugars than proteases and only Neutrase, Celluclast and Viscozyme and Celluclast mixture slightly improved total sugar yield in comparison to the corresponding controls. In another research by Alboofetileh et al. [74] EAE with alcalase had higher fucoidan yield (5.58%) than UAE (3.6%) but combining EAE with UAE resulted in even higher yield (7.87%). Cell wall weakening that occurs during the enzymes hydrolysis treatments could potentially increase the effect of the following extraction process [94]. Extraction yields obtained with 2h alcalase treatment coupled with PLE and PLE alone were not statistically different (*p* > 0.05) while viscozyme treatments coupled with PLE produced lower yields [94].

Research by Hammed et al. [92] revealed that hydrolysis time, extraction stages and enzyme concentration had significant positive effects on sulfated polysaccharides yield. Accordingly, the highest fucoidan yield (25.13%) was achieved under optimum conditions determined at hydrolysis time of 19.5 h, 2 extraction stages and enzyme concentration of 1.5 μl/mL. Regarding the pH effect on total sugar yield, significantly higher concentration was achieved at pH 4.5 compared to pH 6–8 while MWs were significantly lower at pH 4.5 [89].

Alcalase extracted fucoidans had the highest sulfate content and MW, the lowest uronic acid and protein content, while monosaccharide composition remained the same for EAE and CE extracted fucoidans [90]. Protease and carbohydrase enzymes used before extraction, reduced the amount of proteins in alginates below 0.4% [91]. Likewise, Alcalase and Cellulase treatment produced alginates with the lowest chemical contamination with proteins and polyphenols as well as with the lowest MW [93]. EAE reduced MW of the extracted polysaccharides from *Ecklonia radiata* by 20–50% compared to control CE what indicates that enzymes have the ability to hydrolyse certain bonds within fucoidan and alginate molecules [89]. Celluclast led to the highest polysaccharide yield, the highest sulfate content and the lowest protein content compared to the AMG, Viscozyme and Alcalase in sulfated polysaccharide extraction from *Sargassum horneri* [95]. Anticancer and immunomodulatory activities of fucoidan are influenced by higher sulfate content and higher MW which explains why Alcalase treated fucoidan exhibited higher anticancer and immunomodulatory activity [90]. Alginate produced by Alcalase and Cellulase treatment displayed higher DPPH radical scavenging activity and higher reducing power [93] while Celluclast assisted extracts of sulfated polysaccharide from *S. thunbergii* demonstrated the strongest DPPH radical and hydrogen peroxide scavenging activity [96]. Table 5 summarizes the most frequent used parameters of EAE for APS recovery from brown algae.

### 3.3. Purification Procedure

In addition to having variable MWs, monosaccharides composition and sulfate content, extracted APS are usually contaminated with proteins and low molecular weight compounds which were also dissolved in water during extraction [3]. Therefore, they can be additionally purified using different purification methods such as ethanol precipitation, membrane separation, ion-exchange, size-exclusion and affinity chromatographic methods. As it depends on the purity requirements and further function, there are no standard protocols set for purification. In functional food industry ethanol precipitation is the most frequently used method while in scale-up testing membrane separation can be used [3].

Ethanol precipitation is often used as the first step in APS purification, which removes low molecular weight impurities from the polysaccharides. Numerous researchers conducted purification step by adding two or three volumes of ethanol to the extract and allowing the mixture to precipitate overnight at 4 °C [16,42,46,52,54]. Due to shielded oppositely charged groups APS are soluble in solvents with higher dielectric constants such as water. [13]. However, by using solvent with lower dielectric constant, like ethanol, polysaccharides sulfate groups and positive ions will form ionic bonds, which will result in precipitation. Precipitated polysaccharides can therefore be separated from the supernatant by centrifugation. Supernatant with other non-polysaccharide impurities is discarded while precipitate is then dissolved in water and sometimes treated with CaCl_2_ to precipitate alginates. Alginates are then removed by centrifugation, while fucoidan is left in supernatant. Since fucoidan is a macromolecule, supernatant goes through dialysis against water to remove other low molecular weight impurities (laminarin, oligosaccharides and inorganic salts) prior to drying. To isolate fucoidan from the extract January et al. [43] and Sahera et al. [44] used cationic detergent hexadecyltrimethylammonium bromide (CETAVLON or CTAB). In addition, asfucoidan is a sulfated (SO_3_^2-^) polysaccharide, it is negatively charged (polyanion) and may form salts with cationic detergents. These salts are highly insoluble in water and hence they will precipitate. Laminarin and alginate are neutral APS and therefore do not react with cationic detergents and remain water soluble [97].

After isolation fucoidan needs to be dried with one of the various drying methods such as oven drying, vacuum drying, spray-drying and lyophilisation. Selection of drying method depends on the analysis requirements along with potential use of the extracted fucoidan [19]. Although it is quite slow and has relatively low capacity, lyophilisation is usually the preferred method due to its low effect on the fucoidan structure and bioactivity [19]. Furthermore, various combinations of chromatographic techniques can be used to achieve high throughput screening and percent purification. For validation purposes, numerous analytical equipment and instrumental techniques can be used to identify and quantify the active fractions of extracted compounds [98].

## 4. Brown Algae Sulfated Polysaccharides as Drug Delivery Systems and Their Safety

Common commercial applications of APS are mainly related to their colloid properties such as high water-solubility, hydrophilicity and chain aggregation [99]. These properties enable the APS to act as emulsifiers, stabilizers, flocculants as well as gelling, hydrating and thickening agents [100]. APS such as alginate, extracted from brown seaweed, as well as agar and carrageenan from red seaweed, are used by the food industry in numerous food products and beverages adding up to a market value of $10.000 per ton of dry seaweed [101]. As it was shown, alginate and other brown algae sulfated polysaccharides such as fucoidan and laminarin are also being researched for their anti-tumor [102], anti-microbial [103], immunostimulatory [104] and anti-inflamatory [105] activity. In addition, new advances are being made in the research of their drug delivery, tissue engineering and skin rejuvenating properties.

Newly developed active substances are lately calling for innovative, tailor-made drug delivery systems that can secure controlled and targeted drug release. Application of brown APS, due to their excellent gel forming properties, biodegradability and non-toxicity offers many possibilities for the creation of various controlled release matrices, i.e., beads, microparticles, nanoparticles, films etc. [106]. Such polysaccharide matrices are loaded with active ingredients which are, by diffusion and pH related erosion, released in targeted tissues [107]. Release mechanisms of active compounds from different grades of alginate matrices at different acidity were investigated by Sriamornsak et al. [108]. They found all tested alginate salts, except sodium/calcium alginate in acidic and ammonium/calcium alginate in both neutral and acidic medium, can extend drug release period to 8–10 h. In addition, added sodium bicarbonate had influence on acidic environment, which means that it acted successfully as a local pH modifier. On the other hand, calcium acetate can be added to enhance cross-linked gel formation through ionic interactions of alginate G-blocks and calcium cations. These mechanisms thereafter enable brown APS to target the release of active compounds to specific parts of the digestive system, e.g., for gastric or intestinal delivery [109]. Incorporation of compounds such as citric acid, sodium, calcium and zinc salts can also modify the drug release rates of brown APS delivery systems [99,110,111]. Furthermore, drug release targeting can be made more accurate by innovative modulations with chitosan, gelatine and pectin [112,113,114,115,116,117]. These modulations often have superior pH stability and bioadhesive properties and can, e.g., enable the release of active compounds in the colon by biodegrading [118]. Gastroretention can be achieved by the formation of porous systems that are able to keep buoyant in the stomach for up to 24 hours [119,120,121]. In addition, brown algae sulfated polysaccharides drug delivery systems are also being developed for the ophthalmic drugs application and offer enhanced ocular bioavailability as well as adequate hydrogel strength and optical clarity [122,123]. Innovative techniques such as nanoencapsulation are also used to further improve active compounds bioavailability and decrease systemic toxicity [124,125,126]. The variety of drugs for which the delivery can be targeted by brown algae sulfated polysaccharides include antitumor drugs [127], anti-tubercular drugs [128], peptide and protein drugs [129,130], antibiotics [131] etc.

Brown algae sulfated polysaccharides also play very important role in tissue engineering research and development of wound specific healing dressings. Their hydrogels can offer optimal moist environment and oxygen flow while also act as efficient bacterial barriers. They are also highly biocompatible materials and can be easily removed, e.g., via saline irrigation [132]. Lee et al. [133] investigated the wound healing properties of alginate in the treatment of full-thickness skin defects in rats and found significant decrease of wound sizes in alginate treated groups. Fucoidan alone or in combination with chitosan also showed the best reepithelization and fastest wound closure results in rabbit dermal burns [134]. On the other hand, Custódio et al. [135] have shown that laminarin can be used for the formation of biocompatible, mechanically stable and injectable hydrogels that can be used for soft tissue repair. Similarly, alginate and its composites are being researched in the field of bone tissue engineering due to their superior adhesion to cells, biocompatibility and regeneration properties [136]. Besides the physical effects of wound protection, brown APS also possess important pharmacological function in the wound healing process. This role is often attributed to the bioactivity of calcium ions, deriving from calcium alginate, for example [137,138]. In addition, more complex wound healing mechanisms based on the coordinated anti-inflamatory, antioxidative and growth-factor dependant properties of APS are proposed by other authors [133,134]. Additionally, various studies are combining their wound healing activity and drug delivery properties therefore creating active tissue healing accelerators. For this purpose, a variety of pharmacologically active compounds e.g., vitamins, antibiotics, growth factors, etc., are being evaluated [139].

Following the global clean labelling trends, cosmetic industry is recently reducing the use of synthetic chemicals and aiming towards the formulations based on natural sources. Due to remarkable biological activity which includes antioxidative, anti-inflammatory and anti-aging activity as well as depigmenting and UV-shielding effects, brown APS are recently in the focus of numerous studies which deal with their application in skin care products [140]. Wang et al. [141] researched the moisture absorption and retention ability of polysaccharides extracted from various seaweed and found that brown APS had the best results even when compared to hyaluronic acid. Sulfated groups were determined as the main active sites for this ability. Fernando et al. [142] concluded that brown algae sulfated polysaccharides have notable antioxidative and anti-inflammatory properties and are able to inhibit collagenase, elastase and tyrosinase therefore causing anti-wrinkling and skin-whitening effects. Brown algae extracts evaluated in the research of Fitton et al. [143] positively affected skin protection, soothing and spot reduction.

However, much more research is needed to characterize the bioactive content from natural sources such as of brown algae to fully understand their benefits and possible concerns. As yet no reports about toxic effects of marine APS are available and no undesirable effects of overdose and sensitivity to polysaccharides are reported [3]. One of the studies which reported that consumption of fucoidan at the recommended dose (2,000 mg/kg bw/day) did not cause any toxicological indications was performed by Hwang et al. [144]. Neither toxicity nor mutagenicity of fucoidan from *L. japonica* was observed at concentration of 5,000 μg/mL [144] and no toxicity was measured in any blood samples when consuming *F. vesiculosus* extract with 85% fucoidan (300 mg) for 12 weeks [145]. Acute toxicity results by Lim et al. [146] classified *S. binderi* extract as category 5 (unclassified) according to the OECD Guideline since no mortality in rats was observed at the highest dosage (2,000 mg/kg).

## 5. Future Challenges and Potential Industry Application of Brown Algae

The market demand for functional foods enriched with ingredients derived from natural sources is rapidly increasing. Due to the various biological activities, marine APS have a great potential to be used in a wide range of functional foods, cosmeceutical and pharmaceutical products. Development of the standard extraction procedure, including pre-treatment and purification step, appears to be crucial for preservation of the polysaccharides structural integrity and consequently their biological properties. Even though advanced extraction techniques, such as MAE, UAE, PLE and EAE, may display higher extraction efficiencies along with reduced time, cost and energy consumption, their application in marine APS extraction is so far limited to the laboratory research since no research on an industrial scale has been reported. Furthermore, it is highly desirable to develop a simple and reliable method for marine APS structural characterization as it could contribute to the better understanding of their structure-bioactivity relationships which is, regardless of intensive research, not completely clarified.

## Figures and Tables

**Table 1:** The most frequent conventional extraction parameters used for the recovery of brown algae polysaccharides.

Algae	Polysaccharide	PRETREATMENT	EXTRACTION	Purification	Yield	References
		Solvent; Time; Temperature	Solvent; Time; Temperature			
*S. henslowianum*	fucoidan	95% EtOH; 2 × 12 h	H_2_O; 3 × 2 h; reflux	EtOH precipitation; dialysis (12000 Da)	5.1%	[46]
*S. fusiforme*	fucoidan	95% EtOH; 24 h; 30 °C	H_2_O; 3 h; 80 °C	EtOH precipitation; dialysis (3,5 kDa)	3.94–11.24%	[54]
			1.0M HCl; 6 h; 25 °C			
			2% CaCl_2_; 3 h; 50 °C			
*E. maxima* *L. pallida* *S. rugosum*	fucoidan	/	H_2_O; 24 h; 70 °C		/	[43]
			0.15M HCl; 2 h; 65 °C	EtOH precipitation		
		methanol-chloroform-H_2_O (4:2:1); overnight; room temp.	2% CaCl_2_; 5 h; 85 °C	10% CTAB		
*C. barbata*	laminarin	acetone-methanol (7:3); 2 × 24 h; 30 °C chloroform; 2 × 24 h; 30 °C	0.1M HCl; 2 × 2 h; 60 °C	EtOH precipitation; ultrafiltration (50, 10 & 1 kDa)	7.27%	[42]
*Cystoseira compressa*	sodium alginate	acetone; 2 × 24 h; 25 °C methanol; 2 × 24 h; 25 °C	0.1M HCl; 2 h; 60 °C 3% Na_2_CO_3_; 2 h; 60 °C	EtOH precipitation; dialysis (3,5 kDa)	fucoidan—5.2%	[16]
*Dictyopteris divaricata*	polysaccharides	/	H_2_O; 5–7 h; 80–100 °C; water to solid ratio 90–110 mL/g	EtOH precipitation	3.05%	[50]
*Sargassum latifolium*	sodium alginate	/	2% citric acid; 2 h; room temperature 3% Na_2_CO_3_; 1–3 h; 25–45 °C	EtOH precipitation	18.89–40.43%	[52]
*Fucus serratus* *F. vesiculosus* *A. nodosum*	fucoidan	85% EtOH; overnight; room temp.	0.1M HCl; 4 h; 80 °C 1% CaCl_2_; overnight; 4 °C	EtOH precipitation	*F. serratus*—4.2–7.5% *F. vesiculosus*—8.1–12.2% *A. nodosum*—6.5–8.9%	[51]
*D. Membranaceae* *P. Pavonica*	sodium alginate	methanol-dichloromethane (1:1); 3x48h; room temp. petroleum ether; soxhlet acetone; soxhlet	2% CaCl_2_; 3 × 3h 1M Na_2_CO_3_; 2 h	EtOH precipitation	*D. Membranaceae* - 18.93% *P. Pavonica*—66.72%	[45]
*Cystoseira sedoides*	fucoidan sodium alginate	acetone; 24 h; 25 °C 80% EtOH; 24 h; 25 °C 80% EtOH; 24 h; 78 °C	2% CaCl_2_; 7 h; 70°C 2% Na_2_CO_3_; 70°C	dialysis (7 kDa)	fucoidan—4.2% alginate—11%	[15]
*C. myrica*	polysaccharides	petroleum ether acetone	H_2_O; 8 h; 80°C	EtOH precipitation; 10% CTAB; dialysis	5.3%	[44]
*Cystoseira crinite* *C. compressa* *C. sedoides*	fucoidan	methanol-dichloromethane (1:1); 3 × 48 h; room temp.	2% CaCl_2_; 3 × 3h	dialysis (30 kDa)	2.8–3.7%	[55]

**Table 2:** Reported parameters of microwave assisted extraction for extraction of brown algae polysaccharides.

Algae	Polysaccharide	PRETREATMENT	EXTRACTION	Purification	Yield	References
		Solvent; Time; Temperature	Solvent; Time; Temperature			
*F. vesiculosus*	fucoidan	/	H_2_O; 1–31 min; 30, 75, 120 psi 1% CaCl_2_; overnight; 4 °C	EtOH precipitation	1.06–18.22%	[56]
*S. thunbergii*	polysaccharides	/	H_2_O; 15-25 min; 60–80 °C; 500–700 W; water to sample ratio 25:1, 30:1, 35:1 mL/g	EtOH precipitation	2.41–2.75%	[66]
*N. zanardinii*	fucoidan	85% EtOH; overnight; room temp.	H_2_O; 2 × 20 min; 90 °C; 700 W 1% CaCl_2_; 14 h; 4 °C	EtOH precipitation	6.17%	[64]
*A. nodosum*	fucoidan	80% EtOH; 20 h; room temp. 80% EtOH; 5 h; 70°C	0.01M HCl; 15 min; 90 °C 2% CaCl_2_; overnight; 4 °C	EtOH precipitation	5.71%	[65]
*A. nodosum*	fucoidan	80% EtOH; 18 h; room temp. 80% EtOH; 4 h; 70°C	0.01M HCl; 5–30 min; 90–150 °C 2% CaCl_2_; overnight; 4°C	EtOH precipitation	6.48–16.08%	[55]
*S. pallidum*	polysaccharides	/	EtOH (19–27%) and ammonium sulfate (20–24%); 5–25 min; 70–110°C; 600–100 W	dialysis (3000Da); EtOH precipitation	5.6–8.26%	[67]

**Table 3:** Reported parameters of ultrasound assisted extraction for extraction of brown algae polysaccharides.

Algae	Polysaccharide	PRETREATMENT	EXTRACTION	Purification	Yield	References
		Solvent; Time; Temperature	Solvent; Time; Temperature			
*L. hyperborean* *A. nodosum*	laminarin	/	H_2_O and 0.03M HCl; 15 min; 60% amplitude; 20 Hz	EtOH precipitation	5.29–6.24%	[75]
*U. pinnatifida*	polysaccharides	/	0.01N HCl; 3-24 h; 80% amplitude	dialysis	25%	[76]
*F. evanescens*	fucoidan	70% EtOH; 10 days; 23 °C	H_2_O; 23 °C; 5–30 min	ion-exchange chromatography	3.99–4.75%	[77]
*S. witghtii*	fucoidan	/	H_2_O; 30 min; 50% amplitude	EtOH precipitation	14.61%	[70]
*A. nodosum*	fucoidan	80% EtOH; 20 h; room temp. 80% EtOH; 5 h; 70 °C	0.01M HCl; 35 min; 40% amplitude; 20 kHz 2% CaCl_2_; overnight; 4 °C	EtOH precipitation	4.56%	[65]
*N. zanardinii*	fucoidan	85% EtOH; overnight; room temp.	H_2_O; 2 × 20 min; 55 °C; 200 W; 20 kHz 1% CaCl_2_; 14 h; 4 °C	EtOH precipitation	3.6%	[64]
*N. zanardinii*	fucoidan	85% EtOH; 24h; room temp.	H_2_O; 59 min; 70 °C; 196 W; 20 kHz CaCl_2_; overnight; 4°C	EtOH precipitation	3.6%	[74]
*N. zanardinii*	fucoidan	85% EtOH; 24h; room temp.	H_2_O; 40–60 min; 70–90 °C; 100–200 W; 20 kHz 1% CaCl_2_; overnight; 4 °C	EtOH precipitation	3.51%	[71]
*S. binderi* *T. ornata*	alginate	80% EtOH; overnight; room temp.	H_2_O; 30 min; 30–90 °C; 75–150 W; 20 kHz	EtOH precipitation; 5% CaCl_2_	27%	[78]

**Table 4:** Reported parameters of pressurized liquid extraction for extraction of brown algae polysaccharides.

Algae	Polysaccharide	PRETREATMENT	EXTRACTION	Purification	Yield	References
		Solvent; Time; Temperature	Solvent; Time; Temperature			
*N. zanardinii*	fucoidan	85% EtOH; overnight; room temp.	H_2_O; 2 × 10 min; 150 °C; 1500 W 1% CaCl_2_; 14 h; 4°C	EtOH precipitation	13.5%	[64]
*N. zanardinii*	fucoidan	85% EtOH; 24 h; room temp.	H_2_O; 10-30 min; 90–150 °C; 1500 W; 7.5 bar; 20–40 mL/g; 1% CaCl_2_; overnight; 4 °C	EtOH precipitation	4.99–23.77%	[87]
*S. japonica*	fucoidan	/	H_2_O; 0.1% NaOH; 0.1% formic acid; 70% EtOH; 50% EtOH; 25% EtOH; 5 min; 80–200 °C; 5–100 bar; 200 rpm 1% CaCl_2_; overnight; 4°C	EtOH precipitation	8.23%	[82]
*S. japonica*	fucoidan	supercritical CO_2_; 4 h; 50°C; 300 bar	0.1% NaOH; 5–15 min; 100–180 °C; 20–80 bar; 100–300 rpm 0.04–0.09 mg/mL	EtOH precipitation	0.1–12.89%	[85]
*Himanthalia elongata*	polysaccharides	/	H_2_O; 20 min; 100°C	EtOH precipitation; dialysis	15.1%	[81]

**Table 5:** Reported parameters of enzymes assisted extraction for extraction of brown algae polysaccharides.

Algae	Polysaccharide	EXTRACTION	Purification	Yield	Reference
		Enzyme; Concentration; PH; Temperature; Time			
*N. zanardinii*	fucoidan	Alcalase (2.5 mL/g; pH7; 50°C; 24 h)	1% CaCl_2_; overnight; 4°C EtOH precipitation	5.58%	[74]
*S. thunbergii*	sulfated polysaccharide	24h Viscozyme, Celluclast, AMG, Termamyl, Ultraflo, Protamex, Kojijyme, Neutrase, Flabourzyme, Alcalase	EtOH precipitation	18.4–28.3%	[96]
*N. zanardinii*	fucoidan	Alcalase (5% v/v; pH8; 50 °C; 24 h) Celluclast (5% w/v; pH4.5; 50 °C, 24 h) Viscozyme (5% v/v; pH4.5; 50 °C; 24 h) Flavourzyme (5% v/v; pH7; 50 °C; 24 h)	CaCl_2_ - alginates removal EtOH precipitation	Alcalase—5.58% Celluclast—4.80% Viscozyme—4.28% Flavourzyme—4.36%	[90]
*Colpomenia peregrina*	alginates	Alcalase (ِ5% w/w; pH8; 50 °C; 24 h) Cellulase (5% w/w; pH4.5; 50 °C; 24 h)	3% Na_2_CO_3_; pH11; 65°C; 3 h EtOH precipitation	Alcalase—3.8% Cellulase—6.6%	[93]
*Sargassum angustifolium*	alginates	Alcalase (ِ5% w/w; pH8; 50 °C; 24 h) Cellulase (5% w/w; pH4.5; 50 °C; 24 h)	3% Na_2_CO_3_; pH11; 65°C; 3 h EtOH precipitation	Alcalase—3.5% Cellulase—3.47%	[91]
*Turbinaria turbinata*	polysaccharides	cellulase, amyloglucosidase and vicozyme	EtOH precipitation	14–21%	[92]
*S. horneri*	sulfated polysaccharide	24h AMG, Celluclast, Viscozyme, Alcalase	EtOH precipitation	AMG—71.63 Celluclast—88.7% Viscozyme—84.68% Alcalase—81.25%	[95]
*Sargassum muticum*	bioactive compounds	Viscozyme (pH4.5; 50 °C; 2 and 4 h) Alcalase (pH7; 50 °C; 2 and 4 h)	/	13.6–23.5%	[94]
*E. radiata*	carbohydrates	10% v/w; 50 °C; 24 h Viscozyme (pH 4.5); Celluclast (pH 4.5); Ultraflo (pH 7); Alcalase (pH 8); Neutrase (pH 6); Flavourzyme (pH 7)	EtOH precipitation	/	[89]
